# Redistribution of Endosomal Membranes to the African Swine Fever Virus Replication Site

**DOI:** 10.3390/v9060133

**Published:** 2017-06-01

**Authors:** Miguel Ángel Cuesta-Geijo, Lucía Barrado-Gil, Inmaculada Galindo, Raquel Muñoz-Moreno, Covadonga Alonso

**Affiliations:** 1 Department of Biotechnology, Instituto Nacional de Investigación y Tecnología Agraria y Alimentaria, INIA, Ctra. de la Coruña Km 7.5, 28040 Madrid, Spain; mangelcuestageijo@gmail.com (M.Á.C.-G.); barrado.lucia@inia.es (L.B.-G.); galindo@inia.es (I.G.); 2 Department of Infectious Diseases, King’s College London School of Medicine, Guy’s Hospital, London SE1 9RT, UK; 3 Department of Microbiology and Global Health and Emerging Pathogens Institute, Icahn School of Medicine at Mount Sinai, New York, NY 10029, USA; raquel.munoz@mssm.edu

**Keywords:** african swine fever virus (ASFV), ASF viral factory, endosomes, endosomal pathway, phosphoinositides, phosphatidylinositol kinases, microtubules

## Abstract

African swine fever virus (ASFV) infection causes endosomal reorganization. Here, we show that the virus causes endosomal congregation close to the nucleus as the infection progresses, which is necessary to build a compact viral replication organelle. ASFV enters the cell by the endosomal pathway and reaches multivesicular late endosomes. Upon uncoating and fusion, the virus should exit to the cytosol to start replication. ASFV remodels endosomal traffic and redistributes endosomal membranes to the viral replication site. Virus replication also depends on endosomal membrane phosphoinositides (PtdIns) synthesized by PIKfyve. Endosomes could act as platforms providing membranes and PtdIns, necessary for ASFV replication. Our study has revealed that ASFV reorganizes endosome dynamics, in order to ensure a productive infection.

## 1. Introduction

Endocytosis is a common entry pathway for nutrients, receptors and pathogens to get into cells that converges on early endosomes (EE). From EE, cargo can be sorted back to the plasma membrane (PM) through the recycling pathway. Alternatively, it can be directed to the trans-Golgi network or to late endosomes (LE), and finally targeted to lysosomes for degradation [1]. LE is a major cargo-sorting compartment. In contrast, lysosomes are the end-point of the degradative pathway. Endosomal maturation from EE to LE is a dynamic process. Starting on the cytosolic face of the EE, invaginations of the limiting membrane into the lumen of the endosome give rise to the intraluminal vesicles (ILV). Then, EE matures into multivesicular bodies (MVB), and as the pH decreases more ILV are generated [2]. The systematic maturation of this pathway depends on endosomal membrane signaling that is tightly regulated by both proteins and lipids [3]. In fact, the heterogeneity of lipid distribution in endosomal membranes is an organizing principle for the distribution of membrane-associated proteins. Sequential transport from EE to LE involves the switch of GTPase Rab5 to Rab7. Rab proteins and their effectors are recruited by phosphoinositides by specific lipid-binding domains. Short-lived phosphatidylinositol-3-phosphate (PtdIns3P) synthesized by PI3K controls EE functions and is a substrate for the generation of PtdIns(3,5)P_2_ at the LE membrane by the kinase PIKfyve. Other PtdIns, and their respective converting enzymes, are molecular signatures of the PM and the recycling and secretory pathways.

The endocytic pathway ensures a highly dynamic and controlled sorting of cargoes, discriminating the ones that are targeted for degradation from those destined to other cellular locations and functions. Indeed, endocytosis can be exploited by many viruses to infect mammalian cells [4,5,6] and they have adapted to this precise molecular machinery to complete their viral replication cycle successfully.

African swine fever virus (ASFV) is a double-stranded DNA (dsDNA) virus that kills wild and domestic pigs [7,8]. There is currently no effective vaccine, but new experimental approaches to elicit protection have been found [9,10]. From the economic perspective, ASFV is an important pathogen, as it has spread from Africa into east Europe, and currently there is an epidemic outbreak in the Caucasus and eastern European countries [8,11]. African swine fever virus enters the host cells by a complex process involving dynamin and clathrin-mediated and cholesterol-dependent endocytosis [12,13] and macropinocytosis [14,15]. These entry mechanisms, either acting independently or combined, lead the virus to traffic through the endosomal pathway [16]. Endosomal intraluminal acidification [16] and the activity of the GTPase dynamin are the most consistent pre-requisites for ASFV infectivity [13]. Within a few minutes of infection, ASFV is found in LE compartments [16]. Viral decapsidation is the first step of the uncoating process and occurs in the acidic pH of LE. Rab7 is essential for ASFV infection to progress [16]. Then, the cholesterol efflux from endosomes, which is regulated by lipid transporter proteins, is required for further fusion and endosomal exit [17].

Once the uncoated virions are in the cytosol, ASFV replication starts at a single site called viral factory (VF) or viral replication organelle [18] where DNA and proteins accumulate in a microtubule dependent manner. Microtubules (MTs) are cytoskeleton components that are important for ASFV transport [19]. Our group previously reported that ASFV virions interact with dynein motor proteins that move along MTs towards the MT organizing center (MTOC) [19]. Interestingly, the staining pattern of ASFV structural proteins, such as p72 and p54, typically has very strong signals coincident with the virus factory [20]. Additionally, some viral membranes found in VFs can be identified as endoplasmic reticulum (ER) components [20,21,22] and other components such as endosomes and endosomal lipids can also be found in these replication sites. Furthermore, newly-synthesized virions associate with kinesin that drives movement of virions from the VFs to the cell periphery [23,24]. Once there, the new viral progeny is released by budding. Importantly, it has been shown that treatment of cells with MTs depolimerization drugs such as nocodazole, result in the dispersal of ASFV VFs, preventing their correct localization at perinuclear sites [19,25,26]. These facts highlight the dependence of an intact MT network for a successful ASFV infection [19,27].

Since its introduction in Europe through the Caucasus in 2007, African swine fever (ASF) has spread to other neighboring countries, thus threatening porcine production worldwide. Due to the lack of an effective vaccine, ASF control relies on early diagnosis and massive culling of animals. In our aim to discover how ASFV surpasses host cell defenses and reorganizes cellular structures to initiate replication, we started looking for targets occurring at the early stages of infection. ASFV enters host cells by endocytosis, and within a few minutes after infection, viral decapsidation takes place at the acid pH of late endosomes. Then, ASFV exits endosomes to start viral replication and reorganizes endosomal traffic to the perinuclear replication site where VFs are built. Given this, endosomes and endosomal lipid signaling might be adapted by ASFV entering the endosomal pathway to promote replication.

In this work, we have examined the distribution of endosomes, upon ASFV infection, to the viral replication site. These results add to a growing body of evidence pointing out the endosomal membrane and its components as crucial elements at the start of viral replication in several virus models.

## 2. Materials and Methods

### 2.1. Cells, Virus and Infections

Vero cells were purchased from American Type Culture Collection (ATCC) and grown at 37 °C in a 5% CO_2_ atmosphere in Dulbecco’s Modified Eagle’s Medium (DMEM), supplemented with 5% fetal bovine serum (FBS), or 2% for viral infections, containing penicillin/streptomycin (P/S) and Glutamax (G; Gibco, Gaithersburg, MD, USA). Cells were grown on chamber slides (Lab-Tek; Nunc, Roskilde, Denmark), approximately 1.5 × 10^4^ cells/chamber and mock-infected or infected with ASFV-Ba71V isolate [28,29] recombinant ASFV expressing green fluorescent protein (GFP; B54GFP), or cherry fluorescent protein (ChFP; B54ChFP) [21], at a multiplicity of infection (moi) of 1. Both recombinant viruses showed accumulation of the fluorescent GFP or ChFP, expressed as a fusion protein of the viral p54 in the VF, at late times after infection (9–24 h post-infection; hpi). African swine fever virus stocks from culture supernatants were clarified and semi-purified from vesicles by ultracentrifugation at 40,000× *g* through a 40% (*w*/*v*) sucrose cushion in phosphate-buffered saline (PBS) for 1 h at 4 °C. Purified ASFV stocks were sonicated on ice once for 1 min and stored at −80 °C. When synchronization of infection was required, cells were chilled at 4 °C for 20 min, and then, the virus was added. Virus adsorption was performed for 90 min at 4 °C, and after cold PBS washing to remove unbound virus, cells were rapidly shifted to 37 °C with fresh pre-warmed media. YM201636 (YM; Symansis, Cell Signaling Science, Auckland, NZ) was used at 1 µM as a cell permeable inhibitor of the enzyme PIKfyve and was added from 2 h before to 4 h after infection as below described.

### 2.2. ASFV Titration

Viral stocks, or infective ASFV yields from infected samples, were titrated by plaque assay in Vero cells, as previously described [28]. Briefly, preconfluent monolayers of Vero cells in 12-well plates were inoculated with 10-fold sample serial dilutions from samples for 90 min at 37 °C. The inoculum was then removed and 3 mL of semisolid medium was added (1:1 low-melting-point agarose; Gibco), as well as 2× minimal essential medium (MEM; Gibco). Plaque development was visualized after 10–12 days post-infection (dpi), after staining with crystal violet 1% (*w*/*v*) (Sigma-Aldrich, St. Louis, MI, USA).

### 2.3. Detection and Quantitation of the ASFV Genome

The quantitation of the number of copies of ASFV genome was achieved by quantitative real-time PCR (qPCR). The qPCR assay used fluorescent hybridization probes to amplify a region of the p72 viral gene, as described previously described [30]. DNA from cells mock-infected or infected with ASFV Ba71V at an moi of 1 pfu/cell was extracted at 16 hpi and purified with a DNAeasy blood and tissue kit (Qiagen, Hilden, Germany). DNA concentration was measured with Nanodrop. The amplification mixture was prepared on ice as follows: 250 ng template DNA to a final reaction mixture of 20 µL, containing 50 pmol sense and anti-sense oligonucleotides, 5 pmol TaqMan probe and 10 µL PCR Premix Ex Taq (2×; Takara Bio, Clontech, Mountain view, CA, USA) [30]. Positive amplification controls were DNA purified from ASFV virions at different concentrations, as standards. Negative amplification controls consisted of DNA extracted from mock-infected cells. Each sample was included in triplicates, and values were normalized to standard positive controls. Reactions were performed using the ABI 7500 Fast Real-Time PCR System (Life Technologies, Thermo Fisher Scientific, Applied Biosystems, Waltham, MA, USA) with the following parameters: 1 cycle at 94 °C for 10 min, 45 cycles at 94 °C for 15 s and 45 cycles at 58 °C for 1 min.

### 2.4. Proteins Detection by Western Blot

Protein extracts from Vero cells were separated by electrophoresis in 12% acrylamide–bisacrylamide gels, or 7% to detect PIKfyve. Separated proteins were transferred to nitrocellulose membranes, and proteins were detected with specific antibodies in each case. As a secondary antibody, anti-mouse IgG (GE Healthcare, Little Chalfont, UK) or anti-rabbit IgG (Bio-Rad, Oslo, Norway) conjugated to horseradish peroxidase, were used at a 1:5000 dilution. Precision Protein StrepTactin-HRP Conjugate (Bio-Rad) was used to reveal the ladder Precision Plus Protein WesternC (Bio-Rad). As a load control in WB analysis, an anti-mouse antibody against β-tubulin (Sigma) 1:2000 was used. Finally, bands obtained after development with ECL reagent were detected on the Molecular Imager Chemidoc XRSplus Imaging System (Bio-Rad, Hercules, CA, USA). Bands were quantified by densitometry, and data were normalized to control values using Image system software.

### 2.5. Antibodies

Monoclonal antibodies against the virus major capsid protein p72 (Ingenasa, Madrid, Spain) were used at a working dilution of 1:1000 and anti-p30 antibody at 1:100 dilution [31]. African swine fever virus p30 protein is expressed since the initial phases of the infection [32], and p72 and p54 protein are late proteins expressed after viral replication [20]. Both p54 and p72 viral proteins are very abundant in infection and accumulated in the viral replication organelle or VF.

Early endosomes were labeled with anti-mouse EEA1 antibody (BD Biosciences Pharmingen, San Diego, CA, USA), EEA1 being a Rab5 GTPase effector, and anti-rabbit Rab7 (Cell Signaling Technologies, Danvers, MA, USA) was used to label LE both at 1:50 dilution. Multivesicular endosomes (MVE, were labeled with anti-CD63 (Developmental Studies Hybridoma Bank, University of Iowa, clone H5C6), a tetraspanin characteristic of this compartment, at a 1:200 dilution. LY were labeled with anti-Lamp1 (Abcam, Cambridge, UK) at 1:50 dilution. Anti-PIKfyve polyclonal antibody (1:500) was obtained from Abnova (Taoyuan City, Taiwan). The secondary antibodies used were anti-mouse immunoglobulin G (IgG) antibody conjugated to Alexa Fluor 594 and anti-rabbit IgG antibody conjugated to Alexa Fluor 488. Secondary antibodies were purchased from Molecular Probes (Eugene, OR, USA) and diluted 1:200. The specificity of labeling and the absence of signal crossover were determined by examination of single labeled control samples.

### 2.6. Flow Cytometry

At 6 hpi, cells were washed with PBS and harvested by trypsinization. After washing with fluorescence-activated cell sorter (FACS) buffer (PBS, 0.01% sodium azide and 0.1% bovine serum albumin (BSA), cells were fixed and permeabilized with Perm2 (BD Sciences, San Jose, CA, USA) for 10 min at room temperature (RT). Infected cells were detected after incubation with anti-p30 monoclonal antibody (diluted 1:100 in FACS buffer) for 30 min at 4 °C, followed by incubation with phycoerythrin (PE)-conjugated anti-mouse immunoglobulins (1:50, diluted in FACS buffer (Dako, Agilent Tech. Santa Clara, CA, USA) for 30 min at 4 °C. After extensive washing, 10,000 cells per tube in triplicates were scored and analyzed in a FACSCalibur flow cytometer (BD Sciences) to determine the percentage of infected cells under these conditions. The obtained infection rates were normalized to the corresponding control.

### 2.7. Indirect Immunofluorescence, Conventional and Confocal Microscopy

Cells were grown on glass coverslips and fixed in PBS 4% paraformaldehyde (PFA) for 15 min and permeabilized with PBS-0.1% Triton X-100 or saponin (Sigma) for 10 min. Following cell fixation, aldehyde fluorescence was quenched by incubation of cells with 50 mM NH4Cl in PBS for 10 min. After blocking with BSA (Sigma) or normal goat serum (Sigma), cells were incubated with corresponding antibodies and nucleus and were stained with Topro3 (Molecular Probes) before mounting.

Confocal microscopy was carried out in a Leica TCS SPE confocal microscope, using a 63× immersion oil objective. Conventional fluorescence microscopy to analyze Filipin staining was performed in a Leica DM RB microscope, through a 63× immersion oil objective. Image analyses were performed with Leica Application Suite advanced fluorescence (LAS AF Lite) and Image J software version 1.47v for Windows.

### 2.8. Measurement of Rab7 Endosomal Aggregates

The distance of the Rab7 signal to the cellular nucleus in mock- or ASFV-infected cells was carried out with the Image J plug-in “Distance measure”, kindly provided by Dr. Esteban Veiga and Giulia Morlino (Institute for Health Research “Hospital Universitario La Princesa”, CNB, Madrid, Spain).

To measure the distance between two three-dimensional fluorescence distributions in independent channels, we binarized both grey-scale stacks (the plug-in was designed to select two thresholds, thus the region of interest is isolated in both channels). Then, the Euclidean distance between every possible pair of voxels was computed (one in Distribution A and the other in Distribution B). This yields the exact distribution of pairwise distances. Note that the calculation is fully computed in three-dimensions; therefore, it calculates the actual distance between the two regions of interest. Finally, the mean distance was computed after discarding the 5% lower and upper values. This mean is known to be a robust estimate of the distribution mean.

### 2.9. Measurement of Viral Factories and Endosomal Aggregates

The area occupied by VF or endosomal aggregates was measured from immunofluorescence representative images by drawing regions of interest in each picture. It was performed using LAS AF confocal software. Finally, the mean ± *SD* (standard deviation) was calculated from these regions at several time points.

### 2.10. Nocodazole Treatment

Nocodazole was used as a MT depolymerizing drug. Vero cells were seeded and infected at an moi of 1 pfu/cell and treated with 10 µM nocodazole in DMSO 1 hour prior to infection (−1 h), at the time of infection (0 hpi), or 2 and 4 hpi (+2 and +4 hpi). To address the effect of nocodazole in endosome movement in this cell line, we detected acidic endosomes using lysotracker (75 nM), a pH-sensitive dye, for 30 min at 37 °C. Then, confocal images were taken before and after nocodazole treatment and after washing the drug and adding fresh media. Time-lapse microscopy was carried out using a Leica TCS SPE confocal microscope that included a humidified incubation chamber, a CO_2_ controller and a heating unit. Selected stacks were recorded every 10 s using the Leica Microsystems LAS AF program, and the movies were displayed at 1–5 frames per second. Then, 10 µM nocodazole stopped vesicular traffic, and movement was recovered after washing, as it is a reversible drug (data not shown).

### 2.11. Statistical Analysis

Differences between groups were analyzed by the Bonferroni test with GraphPad Prism 6 and Instat 3.05 software for Windows. All experiments were performed in triplicates, and data are presented as mean ± SD of independent experiments. Metrics were normalized to control values and represented in graphics. Asterisks denote statistically-significant differences (*** *p* < 0.001, ** *p* < 0.01 and * *p* < 0.05).

## 3. Results

### 3.1. ASFV Remodels Endosomes

**Figure 1:**
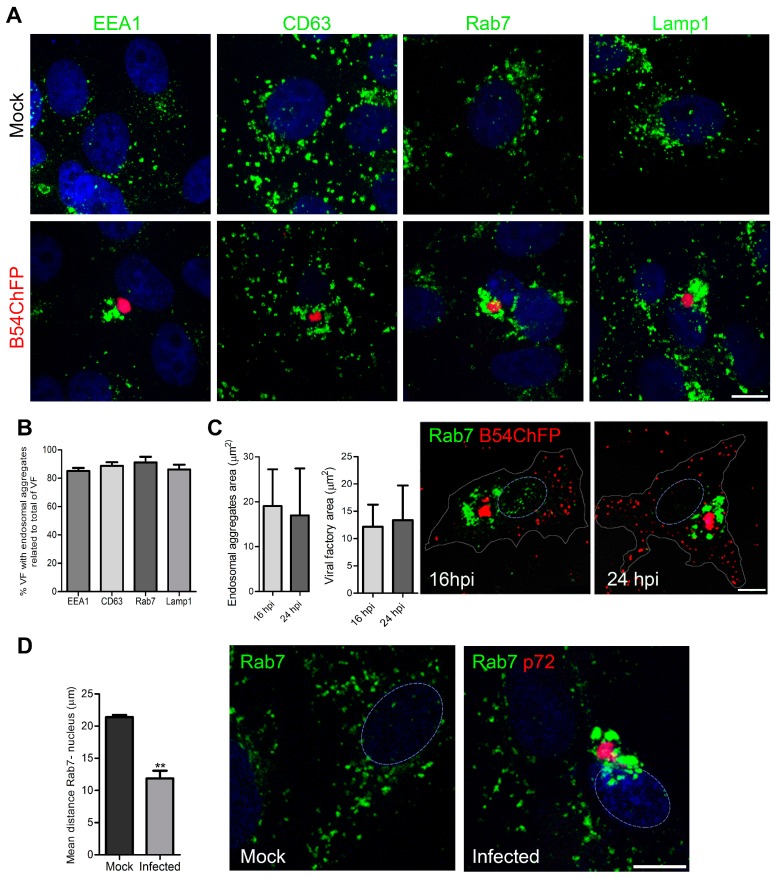
African swine fever virus (ASFV) remodels endosomes. (**A**) Endosome recruitment around the ASFV viral factory (VF) in Vero cells infected with recombinant fluorescent B54ChFP (red) at 16 hpi. Endosome markers are shown in green, on early endosomes (EE; EEA1), multivesicular bodies (MVB; CD63), late endosomes (LE; Rab7) and lysosomes (LY; Lamp1). Above, the typical diffuse cytoplasmic distribution of endosomes in mock-infected cells. Bar 10 µm. (**B**) Percentages of VF with endosome aggregation relative to the total number of VF. (**C**) Cytoplasmic areas occupied by endosomal aggregates or VF at 16 and 24 hpi. Mean ± *SD* from two independent experiments. Bar 10 µm. (**D**) Three-dimensional distances from LE endosomes to the nucleus in control and infected cells at 16 hpi. Mean ± *SD*; *n* = 10 cells in duplicates; significant differences are marked with asterisks (** *p* < 0.01). Bar 10 µm.

Immunofluorescence analysis of the endosomal distribution in ASFV-infected cells showed that ASFV induces a profound change in the vesicular pattern at late time points (10–24 hpi). For this analysis, we used the early endosome marker EEA1, the MVB marker CD63, the LE marker Rab7 and lysosomal marker Lamp1 (Figure 1A), and Vero cells were infected with recombinant ASFV engineered to express GFPs or ChFPs as fusion proteins of p54, as previously described [27], or non-infected.

Between 8 and 16 hpi, the virus establishes its site of replication or VF, which is recognized by confocal fluorescent microscopy as recombinant fluorescent virus accumulated in the perinuclear region. In contrast to non-infected controls, endosomes repositioned around the perinuclear VF in approximately 90% of the VFs in infected cells (Figure 1B). Considerably large areas of aggregated endosomes and VF are depicted in the graphs at 16 and 24 hpi (Figure 1C). Distances to the nucleus of Rab7-expressing vesicles were measured in the *x*, *y* and *z* planes to show that the LE were closer to the nucleus in ASFV-infected cells compared to mock-infected controls (** *p* < 0.01; Figure 1D). Cells with similar sizes were analyzed, and this was obtained when culture conditions were kept constant, and cells were plated at 80% confluence and analyzed at the same time point.

**Figure 2:**
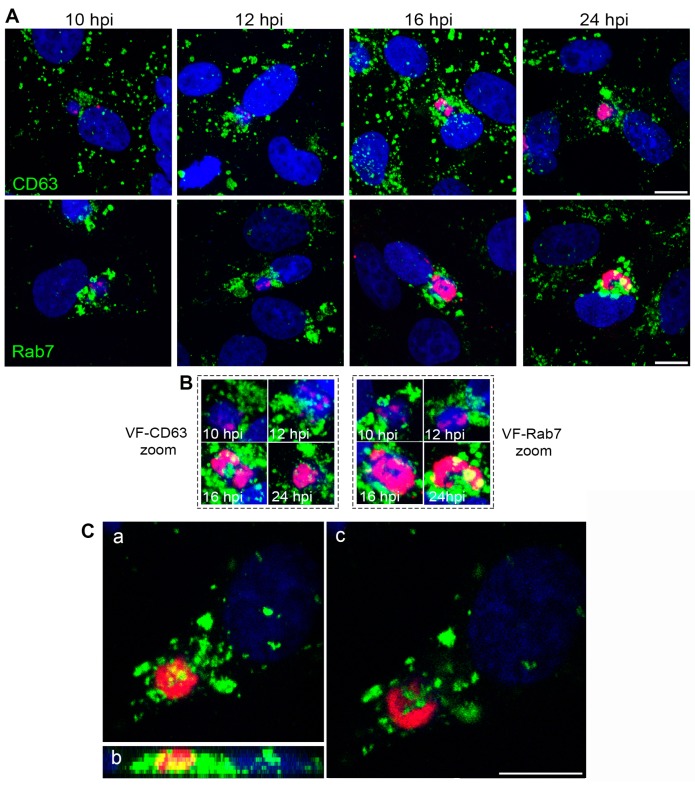
Endosomal membranes participate in the formation of the viral replication organelle. (**A**) VF formation at sequential time points (red; 10–24 hpi). Endosomes are labeled in green (CD63, Rab7), and DNA was stained with Topro3 (blue). Viral DNA and endosomes were first accumulated at the perinuclear area (microtubule (MT) organizing center; MTOC), then dispersed foci of viral proteins appeared intermingled with endosomal membranes colocalizing with viral DNA (pink) or endosomes (yellow). (**B**) Detail of VF is shown in zoom areas for CD63 and Rab7. (**C**) Endosomal membranes arrange together with viral DNA and proteins at the VF. Maximum (a), lateral projections (b), and individual sequential optical planes (c) of ASFV VF, are shown at 16 hpi (B54ChFP; red and Rab7; green). Bars: 10 µm.

The VF that ASFV builds between 8 and 16 hpi consists of a single large cytoplasmic structure with no surrounding membrane located at the perinuclear area where viral replication and morphogenesis occur [7]. We found that the VF was formed in close relationship or interspersed with endosomal membranes (Figure 2A). Endosome clustering occurred in close relationship to the VF as shown in the zoom images (Figure 2B) or sequential optical planes by confocal microscopy (Figure 2C).

### 3.2. Endosomal Recruitment Relies on Viral Infection Progression

**Figure 3:**
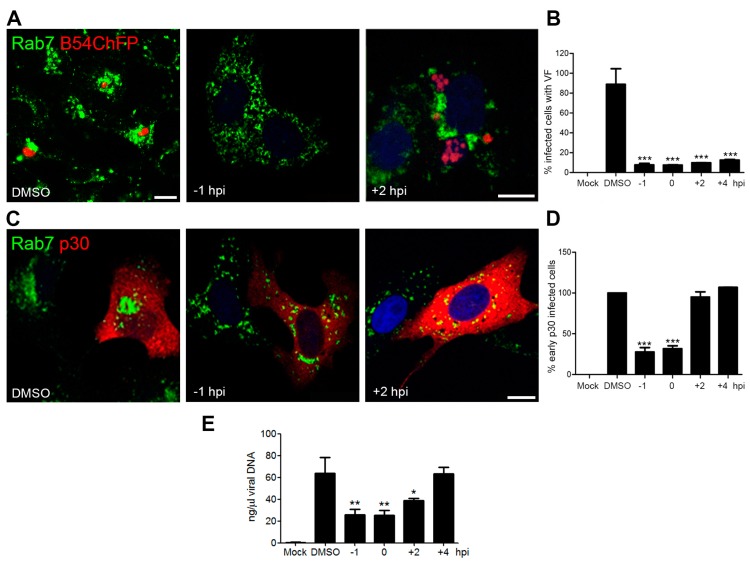
(**A**) The MT cytoskeleton was required for VF formation. MT depolymerization impaired viral replication and endosomal recruitment when nocodazole was added before infection. MT depolymerization later at infection (+2 hpi) reduced the number of cells with VF, and the few visible VF lacked cohesion with smaller endosomal aggregates at 16 hpi. B54ChFP (red) and Rab7 (green). Control cells conserved the characteristic single VF morphology surrounded by aggregated endosomes. Bar: 10 µm. (**B**) Under MT-depolymerizing drug nocodazole treatment, the number of infected cells with VF decreased over 80% as evaluated by fluorescence-activated cell sorter (FACS) analysis at 16 hpi (*** *p* < 0.001). (**C**) Endosome dispersal and early protein p30 expression in cells left untreated, pre-treated with nocodazole or treated after 2 hpi analyzed by confocal microscopy at 16 hpi, using a monoclonal antibody against p30 (red) and an anti-Rab7 (green). (**D**) Pretreatment with nocodazole before infection decreased viral protein p30 expression by FACS analysis at 6 hpi (*** *p* < 0.001). Protein expression was not affected when treatment was started after 2 hpi. (**E**) Viral replication by qPCR in cells left untreated, treated with DMSO, pretreated with nocodazole or treated after 2 and 4 hpi (** *p* < 0.01; * *p* < 0.05).

Given the changes in vesicular distribution, we examined the impact of depolymerizing MT on ASFV endosome repositioning and virus infectivity. Treatment of Vero cells with MT-depolymerizing drug nocodazole effectively inhibited cytoplasmic vesicular traffic labelled with lysotracker. Removal of this drug by adding fresh media restored movement (data not shown). MT depolymerization abolished both VF formation and endosome recruitment when the drug was added at −1 hpi (Figure 3A). Additionally, it inhibited infectivity (*** *p* < 0.001; Figure 3B), expression of viral p30 at 6 hpi (*** *p* < 0.001; Figure 3C,D) and viral replication at 16 hpi (** *p* < 0.01; Figure 3E). This effect was noticeable in cells in which the drug treatment started at 1 h prior to infection. However, when nocodazole was added at infection or at 2 hpi, VF formation (Figure 3B) and viral replication (Figure 3E) were altered, but not p30 expression (Figure 3D).

In the few cells that did become infected under MT depolymerization-initiated post-infection, the viral replication organelle appeared disaggregated (Figure 3A). African swine fever virus VF formation was MT-dependent regardless of the time of nocodazole addition. In contrast, MT depolymerization only affected early p30 protein expression when nocodazole was added before or at the time of infection (Figure 3D). These data suggest that endosomal membrane recruitment occurs after virion transport to the perinuclear area and once early protein expression has taken place, when viral replication starts. Therefore, endosomal recruitment was infection progression dependent.

### 3.3. Endosome Membrane Lipids Are Essential for a Successful ASFV Infection

**Figure 4:**
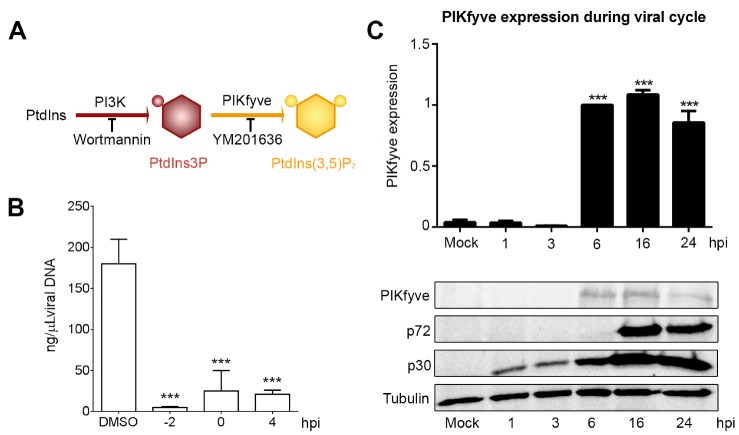
Phosphoinositide interconversion is relevant for ASFV infection. (**A**) Schematics of phosphatidylinositol 3-phosphate (PtdIns3P) and 3,5-biphosphate (PtdIns(3,5)P_2_) interconversions mediated by kinases PI3K and PIKfyve at the endolysosomal membranes. (**B**) Inhibition of PIKfyve converting enzyme activity with YM severely impaired viral DNA replication as shown by qPCR (*** *p* < 0.001). (**C**) PIKfyve expression was upregulated after ASFV infection at the time of viral replication (6–24 hpi). Metrics show the mean ± *SD* in duplicates of WB densitometry related to load control compared to mock-infected cells. Significant differences are marked with asterisks (*** *p* < 0.001).

The dynamic properties of the endosomal membrane are provided by its changing phospholipid composition. PtdIns3P is a substrate for the generation of PtdIns(3,5)P_2_ by the action of the kinase PIKfyve (see the schematics in Figure 4A). Inhibition of PtdIns-converting enzyme PIKfyve with the drug YM (1 µM) from 2 h before infection to 4 hpi as described in Materials and Methods resulted in a significant decrease in viral replication (Figure 4B). Furthermore, PIKfyve enzyme expression increased upon ASFV infection at the time of viral replication (*** *p* < 0.001; Figure 4C).

Inhibition of PtdIns-converting enzyme PIKfyve with the drug YM201636 (1 µM) from 1 h before infection to 4 hpi resulted in a significant decrease in viral replication (Figure 4B). Furthermore, PIKfyve enzyme expression increased upon ASFV infection at the time of viral replication upon ASFV infection (*** *p* < 0.001; Figure 4C).

## 4. Discussion

Our observations demonstrate that ASFV is able to reorganize endosomal traffic to ensure a successful replication. ASFV replication organelle or viral factory (VF) is a single structure lacking an outer limiting membrane in a cytoplasmic location near the nucleus at the MTOC [7]. Fully-formed ASFV VF is surrounded by mitochondria [33,34] and a vimentin cage [35]. We have now described endosomal aggregation at the ASFV replication site. Endosomal membranes are part of early VFs and are found interspersed with the accumulation of newly-synthesized viral DNA and proteins.

It was previously described that membranes used for ASF virion assembly are originated at the ER [22] and membranes close to ASFV particles labelled with antibodies against viral and ER proteins [21,22]. Immunofluorescence analysis shows areas of apparent exclusion of resident ER proteins relative to the rest of the cytoplasm [21,34] where endosomal membranes are accumulated. However, both ER and endosomal membranes could be coincident at the inner part of the VFs in direct contact of areas of viral morphogenesis, which seems possible, but should be considered for further studies.

Viruses hijack several host cell membranes for an efficient replication. Most of these membranes are building elements originated from the secretory pathway, namely ER, Golgi and trans-Golgi [36,37]. However, the presence of endosomal components in a DNA virus assembly site is less frequent. An exception to this is the case of cytomegalovirus (CMV), given that EE markers were found as CMV VF components [38,39]. In the case of RNA virus, endosomes and lysosomes are considered the origin of the cytopathic vacuoles (CPVs) in the *Togaviridae* family (Rubella virus and Semiliki Forest virus). Cytopathic vacuoles are multiple and independent cytoplasmic structures of endo-lysosomal origin entailing a double membrane bilayer containing nascent RNA and viral proteins. Hence, replication and assembly in *Togaviruses* takes place within these modified endo-lysosomes [37]. In contrast, ASFV exits endosomes to complete its replication cycle.

MT-dependence for ASFV infection was previously thought to represent the first transport step of the viral particles to the nucleus [19] and the transport of virions to exit the cell [23,24]. However, our data suggest that intact MTs are also required for viral replication and the VF formation. The absence of MTs resulted in the lack of aggregation of the ASFV VF. Moreover, it impaired endosomal recruitment. Early protein expression only occurred when nocodazole was added before or at the time of infection, but not when it was added at a post-entry stage, suggesting that MTs are also required after virion transport to the perinuclear area. Furthermore, the disaggregation found in VFs under MT depolymerization has been previously reported [19,25,26]. This indicates a crucial role of MTs in the cohesion of the viral replication site.

ASFV reorganizes endosomes to the VFs. Recruitment of endosomes to VFs was dependent on infection progression. MT depolymerization affected endosome recruitment when nocodazole was added before infection, but at late postinfection times (2 hpi) MT depolymerization was unable to inhibit endosomal recruitment completely. ASFV infection progression correlates with endosomal recruitment probably to ensure a successful replication. Whether these events are sequential or simultaneous is still intriguing as the VF is built and therefore should be the subject of further studies.

Endosomal membranes could be a source of lipids that are substrates for replication. We have previously shown that inhibition of PtdIns biosynthesis by PI3K or PIKfyve reduces ASFV production [16]. Now, we found that reduction in viral production by the PIKfyve inhibitor was due to the abolition of ASFV replication. Our results provide further insights in the field of ASFV by demonstrating that the activity of PtdIns-converting kinases is essential for some intracellular pathogens to build their replication sites/niches [40,41]. PI4KIII-converting enzyme that synthesizes PtdIns4P is essential to build the enterovirus replication site and to form the hepatitis C virus membranous web, in both cases by reorganization of the secretory pathway [42,43]. This was shown by detecting modifications of the activity of this enzyme throughout the infective cycle. Furthermore, PIKfyve, responsible for PtdIns(3,5)P2 generation at the LE, participates in the replication of poxvirus [44] as well as in the formation of the replicative vacuole of the intracellular bacteria *Salmonella* [40].

In this study, we found that the inhibition of PIKfyve greatly reduces ASFV replication, and its expression is enhanced upon ASFV replication. In ASFV infection, PIKfyve could exert a similar function as observed in the case of *Salmonella* infection, which also strongly relies on the LE [16]. In conclusion, our results suggest that ASFV replication requires endosomal membranes and PIKfyve enzyme activity.
